# COVID-19 therapies: do we see substantial progress?

**DOI:** 10.1186/s11658-022-00341-9

**Published:** 2022-05-31

**Authors:** Lucyna Matusewicz, Marlena Golec, Aleksander Czogalla, Kazimierz Kuliczkowski, Adam Konka, Joanna Zembala-John, Aleksander F. Sikorski

**Affiliations:** 1 Department of Cytobiochemistry, Faculty of Biotechnology, University of Wrocław, ul. F. Joliot Curie 14a, 50-383 Wrocław, Poland; 2 Silesian Park of Medical Technology Kardio-Med Silesia, ul. M. Curie-Skłodowskiej 10c, 41-800 Zabrze, Poland; 3 Research and Development Centre, Regional Specialist Hospital, Kamieńskiego 73a, 51-154 Wroclaw, Poland; 4 Chair and Department of Medicine and Environmental Epidemiology, Faculty of Medical Sciences in Zabrze, Medical University of Silesia, H. Jordana 19, 41-800 Zabrze, Poland; 5 Acellmed Ltd., M. Curie-Skłodowskiej 10C, 41-800 Zabrze, Poland

**Keywords:** ACE2: coronaviruses, COVID-19, COVID-19 therapies, SARS-CoV-2

## Abstract

The appearance of severe acute respiratory syndrome coronavirus-2 (SARS-CoV-2) and its spread all over the world is the cause of the coronavirus disease 2019 (COVID-19) pandemic, which has recently resulted in almost 400 million confirmed cases and 6 million deaths, not to mention unknown long-term or persistent side effects in convalescent individuals. In this short review, we discuss approaches to treat COVID-19 that are based on current knowledge of the mechanisms of viral cell receptor recognition, virus–host membrane fusion, and inhibition of viral RNA and viral assembly. Despite enormous progress in antiviral therapy and prevention, new effective therapies are still in great demand.

## Introduction

The COVID-19 pandemic, caused by severe acute respiratory syndrome coronavirus-2 (SARS-CoV-2) [1], recently, as of 30 January 2022, resulted in more than 370 million confirmed cases and over 5.6 million deaths globally (https://www.who.int/publications/m/item/weekly-epidemiological-update-on-covid-19---1-february-2022), not to mention unknown long-term or persistent side effects in convalescent individuals.

On 11 February 2020, the International Committee on Taxonomy of Viruses (ICTV) gave the name SARS-CoV-2 to this virus, which was classified in the genus *Betacoronavirus*, family Coronaviridae, order Nidovirales, class Pisoniviricetes, and phylum Pisuviricota. Coronaviruses contain genetic material as a single tight helix of RNA ~ 30 kb in length with positive polarity, ssRNA^+^ [2]. Their nucleocapsid is covered by a lipid bilayer membrane containing proteins and glycoproteins. Electron microscopy showed the crown-like shape of the virus envelope of diameter 80–120 nm, from which the name of the entire group of animal and human viruses is derived. In humans, most coronaviruses (e.g., 229E, OC43, NL63, and HKU1) induce mild infections of the upper respiratory tract and (rather seldom) of the digestive tract. They account for 15–30% of mild, seasonal colds that result from various coronavirus infections [3]. However, at the beginning of this millennium, two severe coronavirus-induced illnesses emerged: one, severe acute respiratory syndrome coronavirus (SARS-CoV), which appeared in China, in Guangdong Province in 2003 and spread to 26 countries and affected 8000 people; the other, Middle East respiratory syndrome (MERS), which started in Saudi Arabia in 2012 and also spread to 27 countries and affected 2500 individuals. Nevertheless, neither of these epidemic events showed pandemic tendencies or exhibited the pandemic character or the major aggressiveness of SARS-CoV-2. It should be noted that even now, over 2 years after the epidemic started, we hope that effective therapy for the disease will appear, as fortunately rapid progress on antiviral drugs has taken place within this period. The best example is the speed of development of antiviral vaccines with modern technologies (e.g., mRNA-based vaccines).

Vaccination is recently ongoing in most countries and, hopefully, by prophylaxis, will attenuate the COVID-19 pandemic. Still, however, owing to technical and financial difficulties and a hardly understandable reluctance to vaccination among certain societies in many countries, a threat of SARS-CoV-2 variants resistant to current vaccine-induced immunity may appear.

Since the beginning of the COVID-19 pandemic, many existing pharmacological treatments have been tested as potential cures for the disease, with better or worse results. Based on the mechanism of action, those substances may be classified into at least eight groups that may inhibit the virus replication cycle at its different stages. According to the European Medicines Agency (EMA), currently there are three authorized COVID-19 treatments: Regkirona (regdanvimab) (12 November 2021), Ronapreve (casirivimab/imdevimab) (12 November 2021) and Veklury (remdesivir) (3 July 2020) (https://www.ema.europa.eu/en/human-regulatory/overview/public-health-threats/coronavirus-disease-covid-19/treatments-vaccines/treatments-covid-19/covid-19-treatments-authorised accessed on 6 December 2021).

There is also a line of potential treatments in the various phases of clinical trials. In February 2022, according to ClinicalTrials.gov, there were ~ 7600 ongoing studies registered, among them 400 at various stages of phase 3 trials (https://clinicaltrials.gov/ accessed in February 2022).

This paper briefly reviews the most critical potential approaches for anti-SARS-CoV-2 therapies.

## SARS-CoV-2 proteins

### Structural proteins

**Fig. 1:**
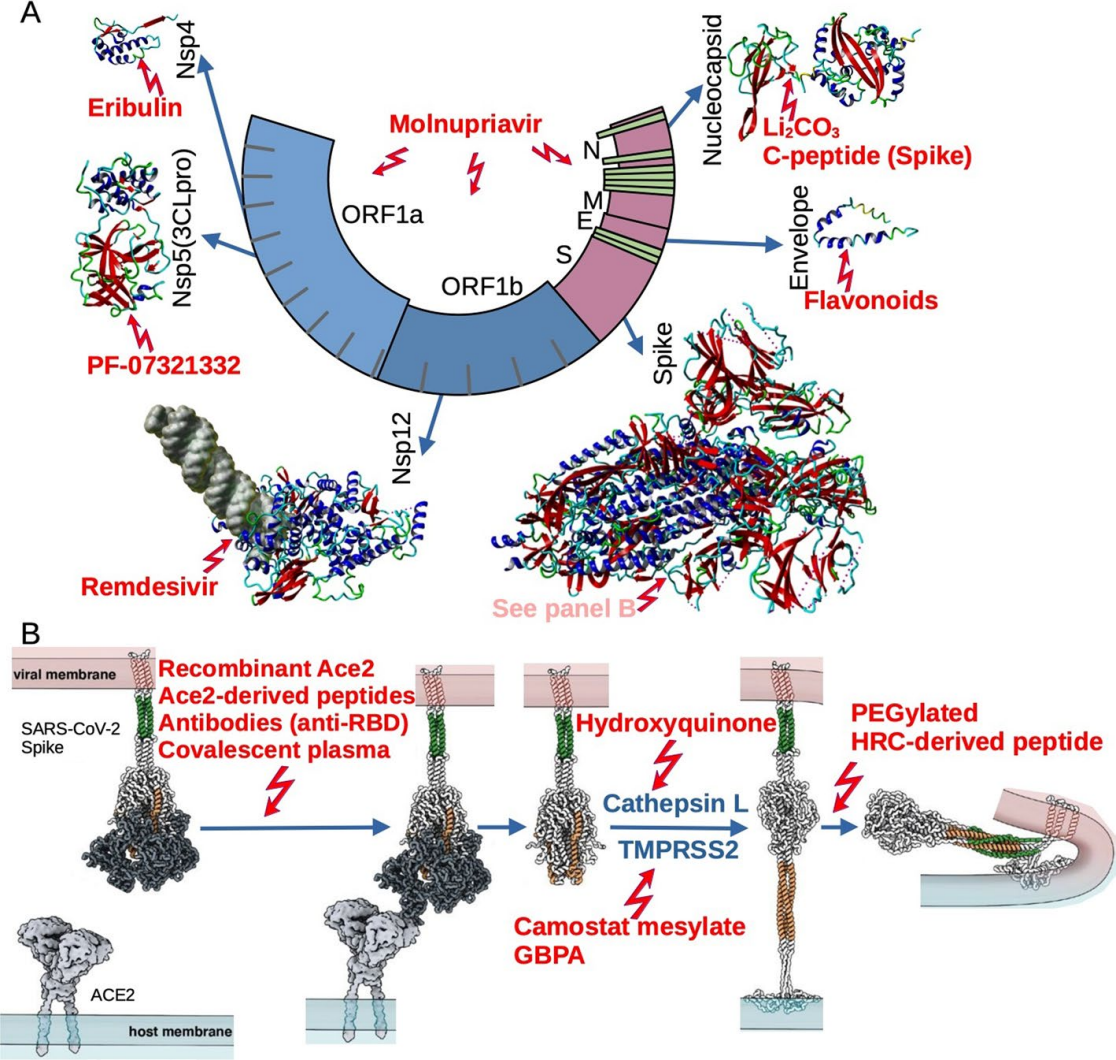
Most promising anti-COVID-19 therapeutics and their molecular targets. **A** Schematic diagram of SARC-CoV-2 genome comprising 14 ORFs encoding structural and nonstructural (Nsp) proteins, which can be targeted by a range of various compounds; structural models were generated according to atomic coordinates accessible in RCSB PDB database: 3GFZ (C-terminal domain of Nsp4 from feline coronavirus), 6LU7 (SARS-CoV-2 main protease complex, Nsp5), 6YYT (SARS-CoV-2 Nsp12 bound to RNA), 6VYB (SARS-CoV-2 spike ectodomain in open state), 5X29 (envelope protein of SARS-CoV), 6VYO, and 6YUN (N-terminal and C-terminal domain on nucleocapsid phosphoprotein from SARS-CoV-2, respectively). **B** Proposed model of interactions between spike protein on the viral membrane and ACE2 on the host cell surface leading to membrane fusion. The process can be inhibited via various compounds at different stages (structural models adapted from [90])

The SARS-CoV-2 genome, the largest RNA viral genome [4] containing 14 open reading frames (ORFs), encodes four important structural proteins among 29 viral proteins in total (Fig. 1A). Major structural proteins encoded by one-third of the viral genome are:

- nucleocapsid protein (N)—the N protein is the only structural protein component of genomic ribonucleoprotein that protects and packages viral RNA. It shows two domains, N-terminal (NTD) and C-terminal (CTD), folding independently, both exhibiting RNA-binding activity [5]. In addition, CTD is responsible for dimerization of this protein [6]. It is also thought to neutralize the host-cell RNA interference (RNAi) antiviral response via binding of double-stranded RNA. It is also a highly immunogenic protein, and therefore considered as a vaccine target candidate protein.
- membrane or matrix protein (M)—the main triple-spanning homo-multimeric 222-amino-acid-residue membrane glycoprotein, which helps to define the shape of the virus. It interacts with proteins S and E to keep the virus in the endoplasmic reticulum–Golgi complex where new virions are assembled and then released via exocytosis. Protein M also interacts with protein N, which suggests participation in the virus packaging process [7].
- envelope (E) protein—a small 76-amino-acid-residue single-pass membrane glycoprotein forming homopentamers. This protein is involved in viral genome release and plays a major role in virus morphogenesis and assembly. It also functions as a viroporin forming nonspecific ion pores in host membranes that are responsible for Ca^2+^ release out of the endoplasmic reticulum (ER) and activation of the inflammasome of the infected organism [8–10].
- spike protein—a large, 1273-amino-acid-residue single-pass trimeric membrane glycoprotein. It is arranged radially on the surface of the virus, which resembles a corona on electron microscopy. Spike protein is responsible for host cell receptor (ACE2) binding and subsequent fusion following proteolysis by host cell proteases. There are two domains in the spike protein:
  - Receptor-binding domain (RBD)—the domain responsible for binding to the receptor.
  - S-NTD, N-terminal galectin-like domain—stabilizing the S2 subunit in the prefusion conformation.

Spike protein plays a crucial role in viral entry to the host cell. It is cleaved into S1 and S2 subunits by host cell protease, mainly furin, during virus maturation in the Golgi system. Upon ACE2 binding, the S2 subunit is cleaved at the S2′ site by TMPRSS2, a host-cell serine protease, which results in the virus entering the cell via fusion. When TMPRSS2 is not available and the virus enters the cell via clathrin-mediated endocytosis, cathepsin hydrolyses this subunit at the S2′ site [11]. Cleavage at the S2′ site results in exposing fusion peptide and dissociation of S1, initiating fusion which allows the target cell to be entered (see also “Drugs inhibiting virus entry to host cells” section).

Several accessory proteins encoded by ORFs 3a, 3b, 6, 7a, 7b, 8b, and 9b and 14 genes located between structural proteins encoding genes are thought to play regulatory roles in viral infection, possibly except for 3a and 7a proteins, which may be absent in the virions.

### Major nonstructural proteins

The entry of the virus to the host cell is followed by uncoating of the genomic RNA. After that, in the cytoplasm of the infected cell, viral gene expression begins. At first, the translation of viral ORF1a and ORF1b, both encompassing two-thirds of the coronavirus genome, produces two large replicase polyproteins, pp1a and pp1ab. Then, from those two polyproteins, 16 nonstructural proteins (nsp1–16) are released upon proteolytic cleavage and posttranslational processing.

The most important functions that direct coronavirus RNA synthesis and processing reside in nonstructural proteins (nsps) 7–16. These nsps are generated by the main viral protease 3CLpro in addition to nsps 1–4 generated by papain-like, cysteine protease, PLpro [12, 13]. The latter is located within multidomain **nsp3** (a 1945-amino-acid-residue protein). 3CLpro, also called Mpro (306 amino acid residues), is in fact **nsp5** and has a cysteine–histidine catalytic dyad at its active site [14]. 3CLpro is crucial in generating polypeptides forming viral transcription machinery, making it an attractive target for developing of low-molecular-weight inhibitors. Other nsps are briefly presented below.

**nsp1**, an 180-amino-acid-residue protein, also called host translation inhibitor, can be regarded as a canonical virulence factor as it suppresses host-cell translation [15] by either binding a 40S ribosomal subunit at the initiation stage or inducing host-cell mRNA degradation [16, 17]. SARS-CoV nsp1 was found to inhibit the interferon (IFN)-mediated innate immune response [18].

**nsp2**, a 638-amino-acid-residue protein, was found to interact with host-cell proteins regulating initiation of translation and endosome vesicle sorting. It is interesting that its C-terminal domain, which is disordered, plays a crucial role in these interactions [19].

**nsp4**, a 500-amino-residue transmembrane glycoprotein that shows the atypical glycosylation signal N–X–C is engaged in the organization of the double-membrane vesicles on which all studied coronavirus nsps are located to form virus replication complexes [20]. An interesting approach was undertaken by Chakraborty et al. Using the available crystal structure of the nsp4 C-terminal domain and molecular docking, they found that, out of 1600 approved drugs, two, eribulin and suvorexant, are promising candidates for repurposing as anti-coronavirus drugs [21] (Fig. 1A).

**nsp6**, a 290-amino-acid-residue protein, was found to bind TBK1 (TANK binding kinase) and inhibit IRF3 (interferon regulatory factor 3) phosphorylation together with other nsps such as nsp13, which inhibits TBK1 phosphorylation, resulting in decreased IFN-I production. Both mentioned proteins have been suggested to suppress IFN signaling via inhibiting STAT1 and STAT2 phosphorylation [22].

**nsp7**, an 83-amino-acid-residue protein, called “copy assistant,” which has α-helical structure resides in the membrane of double-membrane vesicles (DMVs) [23]. nsp7 is a member of the RNA-dependent RNA polymerase (RdRp) complex [24, 25].

**nsp8**, a 198-amino-residue protein, is another “copy assistant” and a member of the RdRp complex. nsp8 is unique to coronaviruses and was reported to have de novo RNA polymerase activity. It therefore has been suggested to function as a primase. It was found to crystallize with nsp7 and nsp12 as tetrameric supercomplex [25, 26] in which catalytic subunit nsp12 binds nsp7–nsp8 dimer and, separately, another nsp8 subunit. Both nsp7 and nsp8 are indispensable for RdRp activity (see below) [24].

**nsp9** is a 113-amino-acid-residue protein that binds a single-stranded RNA (as well as ssDNA) with a *K*_D_ in a micromolar range at unrelated sequences, suggesting that the nucleic-acid-binding activity of nsp9 is not sequence specific. The function of this protein remains unknown. One possibility is that nsp9 preserves nascent RNA from host-cell nucleases [27].

**nsp10** is a 139-amino-acid-residue protein that functions as a methyltransferase (nsp14 and nsp16) stimulator. Inhibiting the methyltransferase activities leads to partial viral RNA capping or lack of capping, making the virus particles susceptible to the host defense system.

**nsp11** is a 13-amino-acid-residue peptide in SARS-CoV-2, but it could be up to 22 residues long in other coronaviruses. Data on the function of this peptide are rather scarce. In the case of other coronaviruses, it is suggested, for example, that mouse hepatitis virus (MHV) nsp10/11 cleavage mutants are not able to replicate [28], while others report that cleavage mutations at nsp10–nsp11–nsp12 of avian infectious bronchitis coronavirus exerted no effect [29]. Sun et al. suggest strong suppression of IFN-I production by overexpressed nsp11 from porcine reproductive and respiratory syndrome virus (PRRSV) [30].

**nsp12**, a 935-amino-acid-residue protein that has RdRp activity. As mentioned above, it forms a complete RdRp complex with nsp8 and nsp7, which serve as a primase and an auxiliary factor respectively. The SARS-CoV-2 nsp12 structure resembles that of SARS-CoV and is conserved across the Coronaviridae family; however, the SARS-CoV-2 counterpart is characterized by lower activity and lower thermostability [25]. The presence of nsp7 and nsp8 is necessary for virus RNA polymerization [31].

**nsp13**, a 601-amino-acid-residue protein is an ATP-driven RNA helicase crucial for SARS-CoV-2 RNA replication [32]. Zn^2+^, Mg^2+^, Mn^2+^, Ca^2+^, or Zn^2+^ are critical in NTPase (ATPase in particular) activity. Evidence coming from some studies points to the importance of nsp13 in the suppression of interferon levels via the sequestration of host deubiquitinase USP13, in particular in the regulation of type I interferon production [33].

**nsp14** is a 523-amino-acid-residue bifunctional enzyme that has two independent activities, namely guanine-*N*^7^-methyltransferase and proofreading exonuclease, and is essential for virus RNA replication. Recent data on MHV suggest that the guanine *N*7-methylation of the 5′ cap mediated by nsp14 contributes to viral resistance of the IFN-I-mediated immune response [34].

**nsp15** is a uridine-specific, 346-amino-acid-residue endoribonuclease that is highly conserved in the coronavirus family. Its enzyme activity resides in the middle and C-terminal domains, while the amino terminal domain is responsible for nsp15 oligomerization. In addition, it is suggested to be involved in interference with the host immune system [35].

**nsp16** is a 298-amino-acid-residue 7-methylguanine-triphosphate-adenosine (m^7^GpppA) specific 2′-*O*-methyltransferase. Both enzymes nsp14 and nsp16 use *S*-adenosylmethionine as a methyl group donor, and both are associated with nsp10. Moreover, as mentioned above, both of these enzymes associate with nsp10 [36].

## Drugs inhibiting attachment of the virus to host cells

### SARS-CoV-2–host cell interaction

SARS-CoV-2, similarly to SARS-CoV, is known to invade the host cells by the viral spike protein that mediates fusion of the viral envelope with the cell membrane, or by exiting the endosome or lysosome also via membrane fusion (Fig. 1B). Angiotensin-converting enzyme 2 (ACE2), which is widely expressed in the cells of lung, intestine, liver, heart, vascular endothelium, testis, and kidney [37], was found to serve as the most prominent receptor of the SARS-CoV viruses [38]. ACE2 is known as an element of the renin–angiotensin system, whose role is to convert angiotensin II (AngII) into angiotensin 1–7 (Ang 1–7) which counteracts the pathophysiologic effects induced by Ang II via its receptors, including vasoconstriction and elevated blood pressure, inflammation, hypercoagulation, and fibrosis. Ang (1–7) peptide functions via the G-protein-coupled receptor MAS [39]. ACE2 is a single-pass integral 805-amino-acid-residue membrane protein, the primary cell-surface receptor for SARS-CoV-2 [1, 40, 41]. Two segments of the SARS-CoV and SARS-CoV-2 host-cell receptor sequence were determined to be located within N-terminal (residues 22–44) and middle (351–357) parts of the molecule [42]. Although the receptor sequence for SARS-CoV is identical to that for SARS-CoV-2 and is rather “tolerant” of substitutions [43], the *K*_D_ values for ACE2 binding of SARS-CoV-2 are about 15–20-fold lower (~ 15 nM) [44, 45]. The structure of the complex of the SARS-CoV-2 receptor binding domain (RBD) and ACE2 was solved with 2.9A resolution [45–47]. The following residues of the receptor (ACE2)-binding motif of SARS-CoV spike protein were found to form a network of interactions with ACE2 residues: Q498, T500, and N501 interact via hydrogen bonding with Y41, Q42, K353, and R357 of the ACE2 molecule, while Q474 and F486 of RBD interact via hydrogen bond and van der Waals forces with ACE2’s Q24 and M82, respectively. Finally, in the N-terminal part of the receptor-binding motif residues, K417 and Y453 are engaged in the interactions with D30 and H34 of ACE2. Although structural features of the complex SARS-CoV-2 RBD with ACE2 are nearly identical to those of SARS-CoV, there are some differences that according to the authors [46] might explain the differences in the affinity of RBD–ACE2 binding. Namely, SARS-CoV-2 RBM contains a four-residue motif (G482–V483–E484–G485) making the ACE2-binding ridge more compact and forming closer contact with the N-terminal helix of human ACE2. Also, stabilization of two virus binding “hotspots” at the RBD–hACE2 interface on the ACE2 molecule occurs, namely K32 and K353, by several residue changes in the RBD.

Another way in which, according to several authors, SARS-CoV-2 may enter host cells is via a protein called neuropilin-1 (NRP-1), which is known to bind furin-digested proteins containing the CendR peptide. Owing to the fact that the cleavage of spike protein by furin leaves the sequence RRAR at the carboxyl terminus of the S1 subunit, the interaction should be feasible [48–50]. It should, however, be noted that, when HEK cells overexpressing NRP-1 were infected with SARS-CoV-2, much lower infection rates were observed compared with ACE2-expressing cells. This is probably related to the rather low affinity of this receptor toward spike protein CendR peptide (*K*_D_ ~ 20 µM) and possibly the fact that other furin-processed ligands are present in the extracellular medium. The data on the presence of the virus in the tissues in which expression of ACE2 is not easily detectable might explain the NRP-1-mediated infection upon high viremia.

Another candidate for SARS-CoV-2 attachment was the phosphatidylserine (PS) receptor AXL belonging to the tyrosine-protein kinase receptor family, which has intermediate affinity (*K*_D_ ~ 1 µM) [51]. However, another study suggests that, although PS receptors alone did not mediate SARS-CoV-2 entry, they may facilitate this process. The authors did not confirm direct interactions between purified spike protein or its N-terminal domain with purified AXL. Therefore, they conclude that AXL interacting with viral PS may be responsible for enhancement of viral infection [52].

During a large interactomic study, two relatively high-affinity receptors, mainly occurring on cells that do not express ACE2 but may undergo SARS-CoV-2 but not SARS-CoV infection, were identified. Krm1 (the first identified Krm, a transmembrane protein containing the kringle domain) and its relative Krm2 were then identified as high-affinity receptors for Dickkopf (Dkk). A ternary complex formed of Krm, Dkk, and Lrp5/6 (the coreceptor of Wnt) suppresses Wnt/β-catenin signaling. In addition, they found specific binding of Krm to RBD of SARS-CoV-2 spike protein at its RBD with a *K*_D_ of ~ 20 nM, which in their hands was 1.5-fold higher than that for ACE2.

Another potential receptor is ASGR1 (C-type lectin, CLEC), a subunit of the asialoglycoprotein receptor, a transmembrane protein that is responsible for recognizing terminal galactose or *N*-acetylgalactosamine residues exposed after sialic acid is lost [53, 54]. This receptor was found to bind RBD with a *K*_D_ of about 95 nM, which is substantially higher than in the case of ACE2 or Krm1. The authors suggest that the monoclonal antibody cocktail ASK composed of blocking antibodies directed against all three (ACE2, ASGR1, and KREMEN1) effectively blocked infection of human lung organoids. However, in addition to the highest affinity toward RBD, ACE2 seems to play the primary role as a SARS-CoV-2 receptor [55]. In summary, this fascinating discovery needs further substantiation and studies on the novel receptor function in SARS-CoV-2 infection.

Several other cell membrane proteins and glycans have been implicated in the SARS-CoV-2–host cell interactions. There is, however, rather little evidence for their function as major receptors, so some of them have been considered as coreceptors.

It is also plausible that viruses can infect host cells through antibody-mediated internalization of virus–antibody immune complexes. Viral particles are recognized and bound through Fab regions of antibody molecules. In contrast, Fc regions interact with the Fc receptor (FcR) of the surface of cells of the host immune system, leading to the formation of a virus–antibody–FcR complex that undergoes endocytosis or phagocytosis. Although this possibility was demonstrated by blocking of FcγR binding (reviewed in [56]), it should be noted that data from animal experiments do not support the possibility of antibody-mediated SARS-CoV-2 infection [57].

### Convalescent plasma

At the beginning of the recent SARS-CoV-2 pandemic, convalescent plasma therapy was often considered a treatment option. In this treatment, plasma is collected from recovered individuals and transfused to patients seriously ill with COVID-19. This type of therapy has been known for a long time; it was successfully applied during other epidemics in the twenty-first century: SARS (2003), H1N1 (2009), MERS (2012), and the Ebola outbreak in Africa (2015; reviewed in [56]). However, this approach seems limited because it includes a small pool of donors, i.e., individuals aged 18–60 years and in good health condition. Also, a reasonable volume (~ 200 ml) of plasma is required for each patient. Numerous preliminary hospital reports on the effectiveness of this therapy have been published (e.g., [58, 59]) (Table 1). However, most recent randomized clinical trials have not confirmed the efficacy of this type of treatment; no difference in the clinical status of patients treated with convalescents’ plasma versus those receiving placebo was observed [60, 61]. There were two extensive clinical trials on the treatment of COVID-19 patients with convalescent plasma. The CONCOR-1 trial (NCT04348656) was conducted in 72 hospitals in Canada, the USA, and Brazil. It was carried out on 940 patients randomized to either convalescent plasma or standard care (ratio 2:1). This study revealed that convalescent plasma did not reduce the risk of intubation or death within 30 days in hospitalized patients with COVID-19. Intubation or death occurred in 32.4% of patients in the treated and 28.0% in the control group [62]. The results of this study are in agreement with those of another extensive clinical trial, RECOVERY (NCT04381936) [63]. In conclusion, the above-mentioned studies clearly demonstrate that treatment with convalescent plasma cannot be considered the standard treatment for patients with COVID-19. It should be noted, however, that one of the studies confirmed its benefits in patients depleted of B cells, treated for B-cell malignancies or autoimmune disease with therapeutic anti-CD20 antibodies (e.g., rituximab), who were unable to mount a humoral immune response [64].

**Table 1:** Treatment regimens with drugs mentioned within the main text, which were used in clinical trials, and the results of these studies

Studied treatment	Targeted population	Dose and time interval	Result	References and clinical trial number
Convalescent plasma	Five critically ill patients with PCR-confirmed SARS-CoV-2 infection and with acute respiratory distress syndrome. Shenzhen, China	Convalescent plasma was transfused to patients between 10 and 22 days after admission. IgG binding titter in plasma was greater than 1:1000, neutralization titer greater than 40	The results of this study show an improvement in the clinical condition of critically ill patients with COVID-19	[59]
	334 hospitalized adult (32.4% female, 67.6% male) patients from Argentina (median age 62) with SARS-CoV-2 infection confirmed by PCR	Convalescent plasma from one or from a pool of two to five donors; transfused volume were 10–15 ml/kg of body weight	No significant difference between groups (convalescent plasma versus placebo) in clinical outcomes	[60], NCT04383535
	103 adults (41.2% women and 58.3% men, median age 70 years) with PCR-confirmed COVID-19 (72 h prior randomization) with severe or life-threatening clinical symptoms. Wuhan, China	Transfusion dose of convalescent plasma 4–13 ml/kg of recipient body weight. Administration of plasma transfusion was 10 ml for the first 15 min, then increased to 100 ml per hour with constant monitoring. Time and units of plasma to transfuse was individually selected	No significant difference in the time to clinical improvement between groups; no significant difference in 28-day mortality or time to discharge	[61], ChiCTR2000029757
	938 adults (≥ 16 years of age in Canada or ≥ 18 years of age in the USA and Brazil), median age 69 years (41% women. 59% men). Confirmed COVID-19 with required oxygen supply	Patients received approximately 500 ml of convalescent plasma from one or two donors. Time and units of plasma to transfuse was individually selected	No difference in the frequency of intubation or death at 30 days of treatment and nontreatment patients	[62], NCT04348656 (CONCOR-1)
	11,558 patients with suspected or laboratory confirmed COVID-19 infection; median age 63.5 years. UK	Patients received two units of convalescent plasma (275 ml) right after randomization and second dose (from different donor) the next day	No significant improvement survival or clinical outcomes in patients hospitalized with COVID-19	[63], NCT04381936 (RECOVERY)
	17 patients depleted of B cells and with prolonged COVID-19 symptoms; median age 58 years; 70% patients were men; SARS-CoV-2 infection confirmed by PCR	Two transfection of two plasma units (200–220 ml each) day after day. Clinical and biological parameters were collected at days 5 and 7 of the study	Convalescent plasma seems to be very promising solution for patients unable to maintain humoral response to SARS-CoV-2	[64]
Casirivimab (REGN10933) and imdevimab (REGN10987)	275 adults with confirmed COVID-19, non-hospitalized; median age 44 years; 51% female and 49% male patients	At day 1—REGN-COV2 on two doses (low dose or high dose) were administrated in 250 ml in 1 h	REGN-COV2 reduced viral load	[69], NCT04425629
Sarilumab	148 adults with moderate-to-severe pneumonia and confirmed COVID-19; France	400 mg of sarilumab was administrated intravenously on day 1; additional 400 mg dose was administrated on day 3 (depending on clinical status and oxygen demand)	Patients with moderate-to-severe COVID-19 pneumonia did not benefit from sarilumab treatment in early outcomes	[71], NCT04324073
Tocilizumab	Adults at the median age 63.3 ± 13.7 years, hospitalized, with viral pneumonia syndrome manifested by, among others, hypoxia and evidence of systemic inflammation 4116 adults of 21,550 patients enrolled into the RECOVERY trial were included in the assessment of tocilizumab, including 3385 (82%) patients receiving systemic corticosteroids. 2022 patients were allocated to tocilizumab and 2094 patients to usual care	Participants were eligible to usual standard of care alone versus usual standard of care plus tocilizumab as a single intravenous infusion over 60 min. The dose of tocilizumab was established by body weight (800 mg if weight was > 90 kg; 600 mg if > 65 and ≤ 90 kg; 400 mg if > 40 and ≤ 65 kg; and 8 mg/kg if weight ≤ 40 kg). A second dose could be given 12–24 h later if the patient’s condition had not improved Outcomes were assessed at 28 days after randomization to tocilizumab versus usual care alone, with further analyses specified at 6 months	In hospitalized COVID-19 patients with hypoxia and systemic inflammation, tocilizumab improved survival and other clinical outcomes. These benefits were seen regardless of the amount of respiratory support and were additional to the benefits of systemic corticosteroids Patients allocated to tocilizumab were more likely to be discharged from hospital within 28 days. Among those not receiving invasive mechanical ventilation at baseline, patients allocated tocilizumab were less likely to reach the composite endpoint of invasive mechanical ventilation or death	[72], NCT04381936 (RECOVERY)
	Adults at the median age 60.9 ± 14.6 years, hospitalized with severe COVID-19 pneumonia confirmed by positive PCR, suffered from hypoxia A total of 438 participants were included in the primary and secondary analyses: 294 in the tocilizumab group and 144 in the placebo group	Patients received a single intravenous infusion of tocilizumab at a dose of 8 mg/kg of body weight or placebo. Approximately one-quarter of the participants received a second dose of tocilizumab or placebo 8–24 h after the first dose The primary analysis was performed at day 28, and the final trial visit occurred at day 60	Under this protocol, the use of tocilizumab did not result in significantly better clinical status or lower mortality than placebo at 28 days	[73], NCT04320615 (COVACTA)
GSK2586881, a recombinant form of human angiotensin-converting enzyme 2 (rhACE2)	Adults between 18 and 80 years diagnosed with acute respiratory distress syndrome (ARDS) who had been mechanically ventilated for less than 72 h A total of 44 participants were randomized for the study: 5 participants took part in part A and 39 in part B, of whom 19 participants received GSK2586881 and 20 participants received placebo	In part A, eligible participants received multiple single intravenous escalating doses of GSK2586881 (0.1 mg/kg, 0.2 mg/kg, 0.4 mg/kg, 0.8 mg/kg) as a slow infusion over 2 days to assess safety, pharmacokinetics, and pharmacodynamics. Following review of data from part A, a randomized, double-blind, placebo-controlled investigation of twice-daily doses of GSK2586881 (0.4 mg/kg) for 3 days was conducted (part B). Participants were followed for up to 7 days	Dose escalation in part A was well tolerated in patients without clinically significant hemodynamic changes The rapid modulation of renin–angiotensin system (RAS) peptides observed in part B suggests target engagement, although the study was not powered to detect changes in acute physiology or clinical outcomes	[77, 78], NCT01597635
Recombinant human angiotensin-converting enzyme 2 (RhACE2 APN01)	Adults at the median age 59 ± 11.9 years, hospitalized with confirmed SARS-CoV-2 infection. Patients whose clinical condition was deteriorating rapidly were not included to the study A total of 178 participants were enrolled in the study: 88 assigned to RhACE2 APN01 and 90 to placebo	Patients were treated with APN01 intravenously twice daily for 7 days. Participants were followed for 28 days to assess safety, with assessments performed on days 3, 7, 10, 14, and 28	Fewer patients treated with APN01 died or received invasive ventilation compared with placebo. Also, reduction in viral load and a tendency to faster recovery in the group treated with APN01 was observed. Treatment with APN01 was safe and well tolerated, and no drug-related severe adverse events were observed during the study	NCT04335136; Apeiron Biologics media release 12 March 2021
Camostat mesylate	Adults with a median age of 62 years (51–75 years), hospitalized with COVID-19 infection defined as PCR-positive for SARS-CoV-2 in respiratory tract samples and hospital admission fo 48 h. Patients unable to understand or sign the informed consent form were not eligible A total of 208 participants were enrolled in the study: 139 assigned to camostat mesylate and 69 to placebo	Two tablets 100 mg camostat mesylate or two placebo tablets were administered orally three times daily (every 8 h) for 5 days Participants were clinically assessed daily until day 5, and at days 14 and 30	Under this protocol, camostat mesilate treatment was not associated with increased adverse events during hospitalization for COVID-19 and did not affect time to clinical improvement, progression to intensive care unit admission, or mortality	[95], NCT04321096
Hydroxychloroquine	Adults at the median age 57 years (44–68 years), hospitalized for less than 48 h with laboratory-confirmed SARS-CoV-2 infection and symptoms of respiratory illness for less than 10 days. The main exclusion criteria were more than one dose of hydroxychloroquine or chloroquine in the prior 10 days A total of 479 participants were enrolled in the study: 242 assigned to hydroxychloroquine and 237 to placebo	400 mg of hydroxychloroquine sulfate in pill form twice a day for the first two doses and then 200 mg in pill form twice a day for the subsequent eight doses, for a total of ten doses over 5 days	Under this protocol, treatment with hydroxychloroquine, compared with placebo, did not significantly improve clinical status at day 14. These findings do not support the use of hydroxychloroquine for treatment of COVID-19 among hospitalized adults	[96], NCT04332991
Hydroxychloroquine + omega-3	A total of 30 adults diagnosed with COVID-19	Intervention group received hydroxychloroquine + 2 g of docosahexaenoic acid (DHA) + eicosapentaenoic acid (EPA) for 2 weeks; control group received hydroxychloroquine	In comparison with control group, patients receiving omega-3 indicated favorable changes in all assessed clinical symptoms except for olfactory. Reducing effects of omega-3 supplementation compared with control group were also observed in the levels of ESR and CRP after treatment. No differences in the liver enzymes serum concentrations were observed between groups after supplementation	[102], IRCT20200511047399N1
Remdesivir	Adults at the median age 58.9 ± 15 years, hospitalized with illness of any duration, with laboratory-confirmed SARS-CoV-2 infection in sample collected for less than 72 h prior to randomization A total of 1062 participants were enrolled in the study: 541 assigned to remdesivir and 521 to placebo	Remdesivir was administered intravenously as a 200-mg loading dose on day 1, followed by a 100-mg maintenance dose administered daily on days 2 through 10 or until hospital discharge or death. A matching placebo was administered according to the same schedule and in the same volume as the active drug Patients were assessed daily during their hospitalization, from day 1 through day 29	Under this protocol, treatment with remdesivir was superior to placebo in shortening the time to recovery in adults who were hospitalized with COVID-19 and had evidence of lower respiratory tract infection (median 10 days as compared with 15 days)	[114], NCT04280705
Molnupiravir	Adults at the median age 40.8 years (18–82 years), non-hospitalized, but with confirmed active SARS-CoV-2 infection in a sample collected ≤ 96 h prior to study entry. Study treatment was expected to begin within ≤ 7 days from first symptom onset. Participants who needed hospitalization or immediate medical attention or those who were vaccinated were excluded from the study A total of 202 participants were enrolled in the study: 140 assigned to molnupiravir in three different doses and 62 to placebo	Molnupiravir was administered orally twice daily for 5 days at dose 200 mg, 400 mg, or 800 mg. A matching placebo was administered according to the same schedule Participants were followed for 28 days to assess safety, with assessments performed on days 1, 3, 5, 7, 14, and 28	Time to viral RNA clearance was decreased in the 800 mg molnupiravir group (median 14 days) compared with the placebo group (median 15 days). Of participants receiving 800 mg of molnupiravir, 92.5% achieved viral RNA clearance compared with 80.3% of placebo recipients by study end. At day 5 of treatment, infectious virus was not isolated from any participants receiving 400 or 800 mg of molnupiravir compared with 11.1% of placebo recipients. Molnupiravir was well tolerated across all doses	[117], NCT04405570
Paxlovid (PF-07321332 and ritonavir)	Adults at the median age 46 years (18–88 years), non-hospitalized, symptomatic, with at least one characteristic condition associated with high risk of progression to severe COVID-19 and with confirmed SARS-CoV-2 infection and symptoms for less than 5 days. The main exclusion criteria were previous confirmed SARS-CoV-2 infection or hospitalization for COVID-19, anticipated need for hospitalization within 48 h after randomization, and prior receipt of convalescent COVID-19 plasma or SARS-CoV-2 vaccine A total of 2246 participants were enrolled in the study: 1120 assigned to PF-07321332 (nirmatrelvir) + ritonavir and 1126 to placebo	Either 300 mg of PF-07321332 (nirmatrelvir) plus 100 mg of ritonavir (a pharmacokinetic enhancer) or placebo were administered orally every 12 h for 5 days (ten doses total) Viral load was analyzed at days 1 (baseline), 3, 5, 10, and 14. Safety was also evaluated	Under this protocol, treatment reduced the risk of hospitalization or death of patients by 89%, compared with placebo, without evident safety concerns The viral load was lower with nirmatrelvir plus ritonavir than with placebo at day 5 of treatment, when treatment was initiated within 3 days after onset of symptoms	[120, 121], NCT 04960202
Lithium (data from retrospective cohort study)	Adults at the average age between 42 and 48 years, treated with lithium for borderline personality disorder (BPD). Patients were considered on lithium treatment if they had an order placed within 90 days prior to their first positive COVID-19 test (COVID-19 cases) or 90 days before their first negative COVID-19 test (COVID-19 controls). To capture long-term use of lithium, patients with two or more lithium orders placed within 12 months before their COVID-19 test were included The data were obtained from three healthcare systems Access to the data of 379,611 cases was obtained, of which 1245 cases were selected for meta-analysis	Patients were prescribed doses effective for the treatment of BPD. As a control, a group that did not use lithium was chosen Patients were tested for COVID-19 via RT-PCR	The analysis of clinical data from over 300,000 patients in three major health systems demonstrates a 50% reduced risk of COVID-19 in patients taking lithium	[124]

### Antibody-based antiviral therapy

Two other approaches were the construction of human antibodies or their variable domains responsible for binding of ACE2 recognition region of spike protein [65, 66], and in using humanized mouse monoclonal antibodies [67] for therapeutic use (Fig. 1B). The authors of both approaches point to the necessity of using antibody mixtures due to the potentially large mutagenic variability of the virus [68].

Casirivimab (REGN10933) and imdevimab (REGN10987) are IgG1 kappa anti-SARS-CoV2 recombinant monoclonal antibodies targeting non-overlapping epitopes of the receptor-binding domain (RBD) of the spike (S) protein which block binding of virus to the cell surface receptor, ACE2. This leads to inhibition of infection of host cells [69]. A randomized clinical trial (NCT04425629) which included 4057 COVID-19 outpatients with one or more risk factors for severe illness in a single treatment with various intravenous doses of REGEN-COV (Ronapreve). A co-packaged combination of two human monoclonal antibodies, casirivimab and imdevimab, showed that REGEN-COV significantly reduced percentage of hospitalizations (71.3% reduced) or death (70.4% reduced) compared with placebo. Casirivimab and imdevimab administered together were well tolerated, rapidly solved symptoms, and had an impact on viral titer load [69] (see Table 1). Combination of these two drugs received its emergency use authorization (EUA) issued by the Food and Drug Administration (FDA) on 2 November 2021. In November 2021, it was approved by the European Medicines Agency (EMA) for the treatment of COVID-19 in adults and adolescents (12 years and older and weighing 40 kg or more) who do not require oxygen therapy and who are at increased risk of for progressing to severe illness resulting in hospitalization (https://www.ema.europa.eu/en/-medicines/human/EPAR/ronapreve, accessed on 1 December 2021) [70].

Tocilizumab and sarilumab are interleukin-6 (IL-6) receptor blockers. Tocilizumab is humanized monoclonal antibody IgG1 against human IL-6 produced in Chinese hamster ovary (CHO) cells by recombinant DNA technology, while sarilumab is a human monoclonal antibody (https://www.accessdata.fda.gov/drugsatfda_docs/label/2017/761037s000lbl.pdf). Interleukin-6, a signal protein produced by the host immune system in response to inflammatory process plays a crucial role in the course of COVID-19. However, one clinical study on sarilumab indicated that it did not improve the condition of patients in the first stages of the moderate-to-severe disease [71] (Table 1). On the other hand, in the clinical trial RECOVERY (NCT04381936), UK participants were hospitalized with COVID-19 with hypoxia and systemic inflammation. Two thousand and twenty-two patients received tocilizumab (400–800 mg, depending on body weight), and 2094 patients were under standard care. The results show that tocilizumab is an effective treatment for hospitalized patients resulting in reduced deaths, shorter time to discharge from hospital, and reduced risk of requiring mechanical ventilation [72] (see Table 1). The EMA started evaluation of tocilizumab (RoActemra) as the therapy of hospitalized adult patients with severe COVID-19 illness who are already under corticosteroids and require extra oxygen treatment or mechanical ventilation (https://www.ema.europa.eu/en/news/ema-starts-evaluating-use-roactemra-hospitalised-adults-severe-covid-19; accessed on 1 December 2021). This trial concluded that tocilizumab improved 90-day survival as well as intensive care unit (ICU) and hospitalization time in critically ill patients. There are also reports of the lack of efficacy of tocilizumab in the treatment of COVID-19; therefore, the doses of the drug used, the selection of the group of patients susceptible to treatment, and the duration of therapy must be carefully analyzed [73] (Table 1). There are also data from clinical trials on positive therapeutic effects of sarilumab (reviewed in [74]).

A very interesting approach to preventing SARS-CoV-2 infection progression was recently published by Park et al. [75]. They obtained a human monoclonal antibody that blocks the virus from attaching to the host-cell receptor, via binding most of the spike protein residues involved in the interaction with ACE2. The authors underlined the therapeutic potential of this antibody and its resistance to the great mutation potential of the coronaviruses. Effectiveness of this antibody was supported in animal experiments on Chinese hamsters.

### ACE2-based virus entry inhibitors (decoy receptors)

Data on the interaction of spike protein with ACE2 were used in the approach to COVID-19 therapy and/or prevention. Using the recombinant human ACE2 as a soluble/nanoparticle-conjugated decoy for SARS-CoV-2 has been considered for some time (Fig. 1B). Some approaches to solve this question have been reported in the literature (for a review, see [76]). Full-length recombinant ACE2 (GSK586881) was found safe in a pilot clinical trial as an infusion drug (from 0.1 to 0.8 mg/kg) to treat acute respiratory distress syndrome [77, 78] (Table 1). It should be noted that the catalytic activity of full-length ACE2 may exert also anti-angiotensin II effect, which would be beneficial to patients with COVID-19 [79].

An important direction of research in this field is engineering of the ACE2 molecule to increase its affinity toward spike protein RBD. One direction is obtaining soluble trimeric ACE2 molecule that interacts with spike protein RBD with a *K*_D_ below 1 nM compared with ~ 15 nM. Cell culture experiments on engineered trimeric ACE2 suggest that it may be a promising antiviral agent to treat COVID-19 [80, 81].

Another direction is a mutational approach to increase affinity towards spike protein RBD. There are many mutational studies on ACE2 (see review [76]) to increase the natural affinity towards spike protein RBD. Deep mutagenesis studies enabled the discovery of mutations of residues exposed at the ACE2-spike protein RBD interaction surface that markedly increased the affinity of this binding. Combining these mutations resulted in variants characterized with affinities toward SARS-CoV-2 comparable to those of monoclonal antibodies [82–84].

The use of recombinant, soluble ACE2 as a drug has proven to be safe and effective in anti-COVID-19 therapy (NCT04335136; Apeiron Biologics media release 12 March 2021; see also Table 1). Further improvement in this solution was constructing the fusion of soluble ACE2 with IgG1-Fc, which made it possible to solve the problem of fast removal of therapeutic protein from circulation [85].

An interesting solution was proposed by Sims et al. [83]. Namely, they demonstrated that expression of increased affinity decoy ACE2 protein in the proximal airway cells when delivered via intranasal administration of an adeno-associated virus vector markedly diminished pathologic consequences of SARS-CoV-2 infection in mouse model.

Direct inhibition of SARS-CoV-2 entry and replication in the oral cavity was proposed to be achieved by using a chewing gum that contained recombinant ACE2 expressed in plant cells. In vitro and human subject studies have indicated both viral particle trapping and cellular entry blocking by microparticle-conjugated ACE2 [86].

A further development is the design of miniproteins that are decoys containing α-helical SARS-CoV-2 binding motifs optimized for high affinity, characterized with a *K*_D_ below 1 nM [87, 88].

In summary, the approach presented above, i.e., using ACE2-based soluble decoy inhibitors, is undoubtedly a promising and potent therapy against COVID-19. These solutions are essentially mutation resistant, as the occurrence of SARS-CoV with higher affinity toward ACE2 would lead to stronger binding of the ACE2 sequence in the decoy drug. If the affinity toward the cellular receptor of the new variant were smaller, it would be less infectious.

## Drugs inhibiting virus entry to host cells

### Fusion inhibitors

As mentioned above, the entry of the SARS-CoV-2 virion into host cells is mediated mainly by spike protein. During one of the last steps of virus replication in infected cells, spike protein in the Golgi apparatus is cleaved by proprotein convertases (e.g., furin) into S1 and S2 subunits. The S1 subunit remains noncovalently associated with the S2 subunit in the mature virion. The S2 subunit that binds ACE2 ensures anchoring to the membrane of the target cell and provides machinery (primarily the fusion domain) necessary to mediate membrane fusion upon infection (reviewed in [11]).

Attachment of spike protein to ACE2 induces conformational changes in both S1 and S2 subunits, which leads to shedding of the S1 subunit and exposure of the “S2′ site,” within the S2 subunit. The S2′ site may be cleaved either by TMPRSS2, a transmembrane serine protease at the cell surface preferred by the SARS-CoV-2 virus-cell entry pathway, or by cathepsin L within endosomes, when ACE2-mediated endocytosis occurs (endosomal entry pathway). The cleavage leads to refolding transition of heptad repeat 1 (HR1), which forms the trimeric HRN/HR1 bundle creating the fusion domain ready to insert into the target cell membrane. Antiparallel interaction of HRC (C-terminal heptad repeat) with HRN (N-terminal heptad repeat) domains of each spike protein trimer forms a stable hexameric structure and brings the viral membrane close to the host cell membrane, facilitating fusion pore formation and viral entry [11, 89] (Fig. 1B).

This mechanism became a basis for studies on a peptide-based pharmaceutical whose mechanism of action relies on the inhibition of fusion and therefore virus entry to host cells. Namely, a group of authors from the Netherlands, the USA, and Italy developed a PEG–lipid conjugate of the peptide derived from the C-terminal heptad repeat (HRC) domain of SARS-CoV-2 spike protein, which was shown to compete with the native sequence and inhibit SARS-CoV-2 fusion with the target cell and therefore the viral infection in vitro and in vivo in experimental animals [90, 91]. Furthermore, invented by the above group of authors, intranasal spray administered daily was shown to prevent SARS-CoV-2 infection of experimental animals when co-housed with infected animals in conditions where 100% of control animals got infected [90].

Among the pharmaceuticals that may inhibit SARS-CoV-2 entry to host cells are also clinically approved protease inhibitors, such as camostat mesylate [92, 93], which is the mesylate salt form of camostat. It is an orally bioavailable, synthetic serine protease inhibitor, with anti-inflammatory, antifibrotic, and potential antiviral activities suggested for COVID-19 treatment. This drug and its metabolite, 4-(4-guanidinobenzoyloxyl)phenylacetic acid (GBPA, FOY 251), are potent inhibitors of a variety of serine proteases and also C1r- and C1 esterases. Along with blocking the activation of trypsinogen in the pancreas, which is known to play a crucial role in the development of pancreatitis, camostat is thought to suppress the expression of numerous cytokines responsible for inflammation and fibrosis of the pancreas. Camostat and its active metabolite GBPA are also known to inhibit the TMPRSS2 and other TMPRSS2-related host-cell serine proteases (among them TMPRSS11D and TMPRSS13). As was mentioned above, these enzymes are thought to mediate viral cell entry of the influenza virus and coronaviruses, thereby suppressing viral infection and replication (Fig. 1B). After encouraging preclinical results [94], the possible application of this drug was tested in a phase II clinical trial [95]. One hundred thirty-seven patients suffering from COVID-19 received camostat mesylate 200 mg three times daily for 5 days, and 68 patients received a placebo. It was demonstrated that, under this protocol, the median time to clinical improvement was 5 days in both camostat mesylate and placebo groups (Table 1). Similarly, treatment with camostat mesylate revealed neither increased adverse events during hospitalization nor increased mortality. It is unknown whether higher drug concentrations would be sufficient to suppress viral entry to cells of the respiratory epithelium.

### Chloroquine and hydroxychloroquine

Another compound that in preclinical studies showed promising outcomes in terms of inhibition of SARS-CoV-19 virus entry to host cells is hydroxychloroquine. It is a derivative of chloroquine in which one of the *N*-ethyl groups of chloroquine is hydroxylated at position 2. Use of hydroxychloroquine, owing to decreased renal and ocular toxicity compared with chloroquine treatment, was approved by the FDA in 1955. Since then, it has been almost exclusively prescribed for oral usage, in the form of tablets. Both drugs are commonly prescribed to treat uncomplicated malaria, rheumatoid arthritis, chronic discoid lupus erythematosus, and systemic lupus erythematosus. As a weak base, chloroquine and hydroxychloroquine lead to a pH increase in endosomes and lysosomes. In this way, these drugs affect protease activities (e.g., above-mentioned cathepsin L), inhibiting cleavage of SARS-CoV-2 spike protein at the S2′ site, which may suppress fusion with the endosomal membrane and viral entry to the cytosol (Fig. 1B). Furthermore, hydroxychloroquine was shown to inhibit the inflammatory response of the organism by blocking the cytokine storm associated with severe progression of COVID-19. Despite promising prognoses, it was proved that the application of hydroxychloroquine alone in the treatment of COVID-19 was not sufficient in the case of hospitalized patients treated with the drug for 14 days (Table 1 and [96]). However, another study indicated that the impaired potency of hydroxychloroquine in COVID-19 treatment might be partially restored in the presence of TMPRSS2 inhibitors [97]. The study suggests a potential benefit of combined hydroxychloroquine and TMPRSS2 inhibitors and proves the lower dependence of SARS-CoV-2 on the endosomal pathway. Indeed, there are many ongoing, more or less promising clinical trials on the combined use of hydroxychloroquine with other drugs, including azithromycin, oseltamivir, favipiravir, zinc, omega-3, ribavirin, and ivermectin (Table 2).

**Table 2:** Combined therapies of hydroxychloroquine with other drugs tested for COVID-19 treatment

Combination of used drugs	Outcome	References
Hydroxychloroquine + favipiravir	Clinical benefits not observed	[99]
Hydroxychloroquine + azithromycin	Clinical benefits not observed	[100, 101]
Hydroxychloroquine + omega-3	Patients receiving omega-3 indicated favorable changes in all clinical symptoms except for olfactory	[102]
Hydroxychloroquine + ribavirin	Clinical benefits not observed	[103]
Hydroxychloroquine + zinc	Zinc supplements did not enhance the clinical efficacy of hydroxychloroquine	[104]

Despite many preclinical studies and clinical trials, there is no drug against COVID-19 available on the market that would effectively inhibit the stage of virus entry into the host cell. In addition, chloroquine is a well-known inhibitor of autophagy that inhibits binding of autophagosomes to vesicles bearing lysosomal enzymes. Autophagy is considered as a part of the innate immunity against viral infections [98].

## Drugs inhibiting replication of the virus

As was briefly mentioned above (see “Major nonstructural proteins” section), a series of proteolytic events concerning polyproteins pp1a and pp1b results in the generation of nsps 1–16, which help to target the host-cell translation machinery to favor viral mRNAs over cellular RNA, assemble viral replication and transcription complex (RTC), and affect innate immunity of the host.

During replication steps, in the perinuclear zone of infected cells appear replication organelles composed of convoluted membranes (CMs), open double-membrane spherules (DMSs), and perinuclear double-membrane vesicles (DMVs). All structures are probably derived from the ER. DMSs were found to be generated during ectopic expression of nsps 3, 4, and 6 [105]. It was found that DMVs are the primary site of coronaviral RNA synthesis [23, 106], while nucleocapsid (N) protein was detectable within CMs and DMS.

Viral RNA is the template for synthesis of either a full-length negative-sense genomic copy that serves as a template for the new generation of positive-sense genomic RNA, or subgenomic negative-sense RNAs, that are used as a template to synthesize a nested set of positive-sense subgenomic RNAs, characteristic for coronaviruses, that are further translated into structural and accessory proteins [107].

It is suggested that DMVs may gather replication machinery and adequate concentrations of molecules necessary for RNA synthesis. It is, however, not clear from the experimental work hitherto carried out whether the assembly of replication machinery takes place inside or outside DMVs [106].

Viral assembly is executed by budding into the ER–Golgi intermediate compartment (EGRIC) [105]. Major viral membrane proteins (M and S) are found in the Golgi membrane, suggesting the use of classical secretory pathway to secrete viral particles. However, it is known that, due to CoV infection, substantial changes in Golgi morphology occur, implying that the virus might use an alternative pathway of secretion. It was suggested that the virus might use a recycling endosome route, skipping Golgi stacks, similarly to other intracellularly budding viruses, lipoproteins, procollagen, and/or protein aggregates [108, 109].

### Remdesivir

One of the drugs whose mechanism of action leads to inhibition of viral replication is remdesivir. Remdesivir is a prodrug (ProTide, prodrug of nucleotide) that after biotransformation in the cell becomes an ATP analog that competes with ATP in RdRp-catalyzed viral RNA synthesis (Fig. 1A). In the case of MERS-CoV, SARS-CoV, and SARS-CoV-2, arrest of RNA synthesis takes place after incorporation of an analog (remdesivir metabolite) followed by three additional nucleotides [110, 111]. It has broad antiviral activity: since 2017, remdesivir has been demonstrated in in vitro studies as effective treatment of infections caused by Arenaviridae, Flaviviridae, Filoviridae (among others, Ebola virus), Paramyxoviridae, Pneumoviridae (among others, respiratory syncytial virus), and Coronaviridae viral families. Although this drug facilitates recovery from COVID-19 and reduces side effects, it requires further study [112], in particular after the WHO Solidarity Trial Consortium found that therapy of COVID-19 hospitalized patients with remdesivir, similarly as with hydroxychloroquine, lopinavir, and interferon, had no or little effect, as evidenced by overall mortality, initiation of ventilation, and duration of hospitalization [113]. However, further studies and clinical trials (e.g., [114], see Table 1) led to the FDA’s decision to grant remdesivir emergency use authorization (EUA) for the treatment of hospitalized patients with severe COVID-19 in May 2020 [Coronavirus (COVID-19) Update: FDA Issues Emergency Use Authorization (EUA) for Potential COVID-19 Treatment. 05/01/2020. Available at: https://www.fda.gov/news-events/press-announcements/coronavirus-covid-19-update-fda-issues-emergency-use-authorization-potential-covid-19-treatment], and in October 2020, remdesivir was the first, and remains the only, FDA-approved treatment for COVID-19 patients requiring hospitalization (https://www.fda.gov/news-events/press-announcements/coronavirus-covid-19-update-fda-issues-emergency-use-authorization-potential-covid-19-treatment).

### Molnupiravir

Another extensively investigated COVID-19 treatment drug that may disrupt the replication process is molnupiravir (MK-4482, EIDD-2801). It is an orally bioavailable isopropylester, a prodrug of N4-hydroxycytidine. The active form of the drug is incorporated into the genome of RNA viruses, leading to the accumulation of mutations that finally lead to inhibition of virus replication [115] (Fig. 1A). The drug was initially developed against the influenza virus; however, it was repurposed as an orally efficacious COVID-19 therapeutic with success in ferrets [116] and subsequently in humans (117; clinical trial number NCT04405570; Table 1). Merck announced the phase 3 clinical study (MOVe-OUT), which indicates at least 50% efficiency in reducing the risk of hospitalization or death compared with the placebo control. Therefore, the FDA issued an EUA for molnupiravir in December 2021. However, the EUA was only narrowly approved because of concerns over the efficacy and potential mutagenic effects resulting in new variants of the virus [115] (FDA News Release https://www.fda.gov/news-events/press-announcements/coronavirus-covid-19-update-fda-authorizes-additional-oral-antiviral-treatment-covid-19-certain).

### PLpro and 3CLpro inhibitors

Another identified potent antiviral drug target is SARS-CoV-2 PLpro (nsp3), which is essential for SARS-CoV-2 replication and is also suggested to play an important role in the innate immune response to viral infection analogous to SARS-CoV. This occurs via inhibition of the synthesis of cytokines and chemokines responsible for activation of the host’s innate immune response against viral infection [13, 118, 119]. Inhibitors of this protease are potent anti-SARS-CoV-2 drug candidates (for review, see, e.g., [13]), but they still require further study.

Recently, Pfizer announced the results of interim protocol from a phase 2/3 study of the oral therapeutic Paxlovid, which is a mixture of PF-07321332, an inhibitor of 3CLpro, and a low dose of ritonavir, a generic HIV drug that boosts the effectiveness of protease inhibitors [120] (Fig. 1A). This drug also received an emergency use authorization (EUA) from the FDA in late 2021. Using 3CLpro as a target for a mechanism of action of an anti-COVID drug seems very promising: 3CLpro resides in nsp5 and, as mentioned above (“Major nonstructural proteins” section), releases the majority of nsps from the polyproteins. Furthermore, 3CLpro is highly sequence-specific; thus, compounds targeting this protease have little or no impact on host cellular proteases. The trial results showed that the application of this drug in non-hospitalized patients reduced the risk of hospitalization or death by 89% compared with placebo [121] (clinical trial number NCT04960202; see also Table 1). This drug was designed for oral administration at the first stage of infection or after presumed exposure to help patients avoid severe illness course and its consequences, i.e., hospitalization or even death.

## Drugs inhibiting assembly and release of the virus, targeting structural proteins other than spike protein

Coronaviruses bud within the endoplasmic reticulum–Golgi intermediate compartment (ERGIC), from where they acquire their membrane envelope, which makes them distinct from other well-studied enveloped viruses [122]. It has been well known that the delivery of these viruses from their sites of formation (ERGIC) to the cell exterior occurs via a constitutive secretory pathway involving the Golgi compartment. However, since infection of cells by these viruses is accompanied by the disruption of normal Golgi system morphology and, moreover, blocking the Golgi-based secretory pathway did not seem to affect the amount of virus being released [108, 123], there are suggested alternative ways of coronavirus secretion, among them non-Golgi pathways, related to recycling endosomes or lysosomes.

Taking into account functions of the structural proteins involved in virus assembly, it is apparent that they are a promising target for a novel group of anti-COVID drugs. Indeed, the outcomes of one of the recently published retrospective studies indicated that the use of lithium (mostly in the form of lithium carbonate contained in the prescription drugs), an inhibitor of glycogen synthase kinase 3 (GSK-3), an enzyme that phosphorylates viral protein N, had a significantly reduced risk of COVID-19 [124] (Fig. 1A). The same study showed that phosphorylation of protein N by GSK-3 is crucial for its function and that the SARS-CoV-2 protein N contains GSK-3 consensus sequences, conserved in various coronaviruses, raising the possibility that the virus may also be sensitive to other GSK-3 inhibitors.

Another study demonstrated that protein E might also be a promising target for anti-COVID drugs. It was shown that some flavonoids, in particular epigallocatechin and quercetin, as well as rimantadine and 5-(*N*,*N*-hexamethylene)amiloride (HMA), may serve as inhibitors of the protein E channel [125] (Fig. 1A).

Finally, it was demonstrated that disruption of the interactions between proteins S and N might also lead to inhibition of virus replication in vitro [126]. It appeared that peptides derived from the C-terminus of spike protein inhibited MERS-CoV and SARS-CoV-2 replication (Fig. 1A).

## Final remarks

As mentioned above, there is no dedicated drug for COVID-19. Currently, over 7000 clinical trials on COVID-19 are conducted underway worldwide. Those trials concern drugs, diagnostics, observations, etc. In our brief review, we tried to summarize currently discussed therapies for COVID-19 considering all the stages of the viral host cell entry and replication that can be pharmacologically inhibited. Those drugs alone or in combination could help systematize available and tested treatments for SARS-CoV-2.

One has to keep in mind that a large number of compounds and a large number of therapeutic approaches together with the mentioned repurposed drug are under various stages of studies (for reviews, see, e.g., [127, 128]).

We have to mention the other approaches that have not found applications as repurposed drugs or are only the basis for future therapy development, such as modeling of metabolic processes taking place in human lung cells upon SARS-CoV-2 infection, that enabled the identification of 12 bioactive molecules that inhibit ten enzymes of lipid and carbohydrate metabolism as well as protein palmitoylation, the most promising being Triacsin C and Celgosivir [129]. Another possibility is addressed by authors suggesting inhibition of glycosylation by tunicamycin, which is a mixture of homologous nucleoside antibiotics that inhibits the eukaryotic polyprenol-P-glycosylating phosphotransferases, including GlcNAc phosphotransferase, which catalyzes the first step of NXS/T-attached glycan synthesis in the ER. Tunicamycin is suggested as a possible anticancer drug evoking the ER stress and apoptosis in studied cancer cell lines (see, e.g., [130]). Proliferation of the SARS-CoV-2 in the presence of tunicamycin was shown to result in the inhibition of glycosylation of viral proteins and complete lack of spike protein giving spikeless virions [131].

An important aspect of virus–host relation during SARS-CoV-2 infection and COVID-19 course is the role of miRNAs. This issue has been a subject of recent reviews [132, 133]. One of the first known major modes of action of the virus on the host cell is sequestering of the host miRNAs [134]. These aspects may also become bases of future therapies.

## Conclusion

In summary, as mentioned above (by no means complete), a multidirectional approach to the anti-COVID-19 therapy undertaken by many research and industrial centers worldwide, in our opinion, will hopefully help to control the disease.

## Data Availability

Not applicable.

## Abbreviations

BPD: Borderline personality disorder
CLEC: C-type lectin
COVID-19: Coronavirus disease 2019
CTD: C-terminal domain
DMVs: Double-membrane vesicles
EGRIC: ER–Golgi intermediate compartment
EMA: European Medicines Agency
EUA: Emergency use authorization
FcR: Fc receptor
FDA: US Food and Drug Administration
GBPA: 4-(4-Guanidinobenzoyloxyl)phenylacetic acid
GSK-3: Glycogen synthase kinase 3
hACE2: Human angiotensin-converting enzyme 2
HEK: Human embryonic kidney
HMA: 5-(*N*,*N*-Hexamethylene)amiloride
HR: Heptad repeat
HRC: C-terminal heptad repeat
HRN: N-terminal heptad repeat
IL: Interleukin
*K*_D_: Equilibrium dissociation constant
m7GpppA: 7-Methylguanine-triphosphate-adenosine
MERS: Middle East respiratory syndrome
MHV: Mouse hepatitis virus
NRP-1: Neuropilin-1
nsp: Nonstructural proteins
NTD: N-terminal domain
ORF: Open reading frame
PRRSV: Porcine reproductive and respiratory syndrome virus
PS: Phosphatidylserine
RBD: Receptor-binding domain
RdRp: RNA-dependent RNA polymerase
RTC: Replication and transcription complex
SARS-CoV: Severe acute respiratory syndrome coronavirus
SARS-CoV-2: Severe acute respiratory syndrome coronavirus-2
TMPRSS2: Transmembrane protease, serine 2

## References

[1] Hu B, Guo H, Zhou P, Shi ZL (2021). Characteristics of SARS-CoV-2 and COVID-19. Nat Rev Microbiol.

[2] V'Kovski P, Kratzel A, Steiner S, Stalder H, Thiel V (2021). Coronavirus biology and replication: implications for SARS-CoV-2. Nat Rev Microbiol.

[3] Fung TS, Liu DX (2019). Human coronavirus: host–pathogen interaction. Annu Rev Microbiol.

[4] Woo PC, Huang Y, Lau SK, Yuen KY (2010). Coronavirus genomics and bioinformatics analysis. Viruses.

[5] Chang CK, Hou MH, Chang CF, Hsiao CD, Huang TH (2014). The SARS coronavirus nucleocapsid protein—forms and functions. Antivir Res.

[6] Peng Y, Du N, Lei Y, Dorje S, Qi J, Luo T (2020). Structures of the SARS-CoV-2 nucleocapsid and their perspectives for drug design. EMBO J.

[7] Neuman BW, Kiss G, Kunding AH, Bhella D, Baksh MF, Connelly S (2011). A structural analysis of M protein in coronavirus assembly and morphology. J Struct Biol.

[8] Nieto-Torres JL, Verdiá-Báguena C, Jimenez-Guardeño JM, Regla-Nava JA, Castaño-Rodriguez C, Fernandez-Delgado R (2015). Severe acute respiratory syndrome coronavirus E protein transports calcium ions and activates the NLRP3 inflammasome. Virology.

[9] Weiss SR, Navas-Martin S (2005). Coronavirus pathogenesis and the emerging pathogen severe acute respiratory syndrome coronavirus. Microbiol Mol Biol Rev.

[10] Nakauchi M, Kariwa H, Kon Y, Yoshii K, Maeda A, Takashima I (2008). Analysis of severe acute respiratory syndrome coronavirus structural proteins in virus-like particle assembly. Microbiol Immunol.

[11] Jackson CB, Farzan M, Chen B, Choe H (2022). Mechanisms of SARS-CoV-2 entry into cells. Nat Rev Mol Cell Biol.

[12] Harcourt BH, Jukneliene D, Kanjanahaluethai A, Bechill J, Severson KM, Smith CM (2004). Identification of severe acute respiratory syndrome coronavirus replicase products and characterization of papain-like protease activity. J Virol.

[13] Rut W, Lv Z, Zmudzinski M, Patchett S, Nayak D, Snipas SJ (2020). Activity profiling and crystal structures of inhibitor-bound SARS-CoV-2 papain-like protease: a framework for anti-COVID-19 drug design. Sci Adv.

[14] Fan K, Wei P, Feng Q, Chen S, Huang C, Ma L (2004). Biosynthesis, purification, and substrate specificity of severe acute respiratory syndrome coronavirus 3C-like proteinase. J Biol Chem.

[15] Lei L, Ying S, Baojun L, Yi Y, Xiang H, Wenli S (2013). Attenuation of mouse hepatitis virus by deletion of the LLRKxGxKG region of Nsp1. PLoS ONE.

[16] Huang C, Lokugamage KG, Rozovics JM, Narayanan K, Semler BL, Makino S (2011). SARS coronavirus nsp1 protein induces template-dependent endonucleolytic cleavage of mRNAs: viral mRNAs are resistant to nsp1-induced RNA cleavage. PLoS Pathog.

[17] Thoms M, Buschauer R, Ameismeier M, Koepke L, Denk T, Hirschenberger M (2020). Structural basis for translational shutdown and immune evasion by the Nsp1 protein of SARS-CoV-2. Science.

[18] Banerjee AK, Blanco MR, Bruce EA, Honson DD, Chen LM, Chow A (2020). SARS-CoV-2 disrupts splicing, translation, and protein trafficking to suppress host defenses. Cell.

[19] Mompeán M, Treviño M, Laurents DV (2021). Partial structure, dampened mobility, and modest impact of a His tag in the SARS-CoV-2 Nsp2 C-terminal region. Eur Biophys J.

[20] Perlman S, Netland J (2009). Coronaviruses post-SARS: update on replication and pathogenesis. Nat Rev Microbiol.

[21] Chakraborty J, Maity A, Sarkar H (2021). A systematic drug repurposing approach to identify promising inhibitors from FDA-approved drugs against Nsp4 protein of SARS-CoV-2. J Biomol Struct Dyn.

[22] Xia H, Cao Z, Xie X, Zhang X, Chen JY, Wang H (2020). Evasion of type I interferon by SARS-CoV-2. Cell Rep.

[23] Gosert R, Kanjanahaluethai A, Egger D, Bienz K, Baker SC (2002). RNA replication of mouse hepatitis virus takes place at double-membrane vesicles. J Virol.

[24] Perry JK, Appleby TC, Bilello JP, Feng JY, Schmitz U, Campbell EA (2021). An atomistic model of the coronavirus replication–transcription complex as a hexamer assembled around nsp15. J Biol Chem.

[25] Peng Q, Peng R, Yuan B, Zhao J, Wang M, Wang X (2020). Structural and biochemical characterization of the nsp12–nsp7–nsp8 core polymerase complex from SARS-CoV-2. Cell Rep.

[26] Gorkhali R, Koirala P, Rijal S, Mainali A, Baral A, Bhattarai HK (2021). Structure and function of major SARS-CoV-2 and SARS-CoV proteins. Bioinform Biol Insights.

[27] Egloff MP, Ferron F, Campanacci V, Longhi S, Rancurel C, Dutartre H (2004). The severe acute respiratory syndrome-coronavirus replicative protein nsp9 is a single-stranded RNA-binding subunit unique in the RNA virus world. Proc Natl Acad Sci USA.

[28] Deming DJ, Graham RL, Denison MR, Baric RS (2007). Processing of open reading frame 1a replicase proteins nsp7 to nsp10 in murine hepatitis virus strain A59 replication. J Virol.

[29] Fang SG, Shen H, Wang J, Tay FP, Liu DX (2008). Proteolytic processing of polyproteins 1a and 1ab between non-structural proteins 10 and 11/12 of coronavirus infectious bronchitis virus is dispensable for viral replication in cultured cells. Virology.

[30] Sun Y, Ke H, Han M, Chen N, Fang W, Yoo D (2016). Nonstructural protein 11 of porcine reproductive and respiratory syndrome virus suppresses both MAVS and RIG-I expression as one of the mechanisms to antagonize type I interferon production. PLoS ONE.

[31] Kirchdoerfer RN, Ward AB (2019). Structure of the SARS-CoV nsp12 polymerase bound to nsp7 and nsp8 co-factors. Nat Commun.

[32] Jang KJ, Jeong S, Kang DY, Sp N, Yang YM, Kim DE (2020). A high ATP concentration enhances the cooperative translocation of the SARS coronavirus helicase nsP13 in the unwinding of duplex RNA. Sci Rep.

[33] Guo G, Gao M, Gao X, Zhu B, Huang J, Luo K (2021). SARS-CoV-2 non-structural protein 13 (nsp13) hijacks host deubiquitinase USP13 and counteracts host antiviral immune response. Signal Transduct Target Ther.

[34] Pan R, Kindler E, Cao L, Zhou Y, Zhang Z, Liu Q (2022). N7-Methylation of the coronavirus RNA cap is required for maximal virulence by preventing innate immune recognition. MBio.

[35] Kim Y, Wower J, Maltseva N, Chang C, Jedrzejczak R, Wilamowski M (2021). Tipiracil binds to uridine site and inhibits Nsp15 endoribonuclease NendoU from SARS-CoV-2. Commun Biol.

[36] Bobrovs R, Kanepe I, Narvaiss N, Patetko L, Kalnins G, Sisovs M (2021). Discovery of SARS-CoV-2 Nsp14 and Nsp16 methyltransferase inhibitors by high-throughput virtual screening. Pharmaceuticals.

[37] Hamming I, Timens W, Bulthuis ML, Lely AT, Navis G, van Goor H (2004). Tissue distribution of ACE2 protein, the functional receptor for SARS coronavirus. A first step in understanding SARS pathogenesis. J Pathol.

[38] Li W, Moore MJ, Vasilieva N, Sui J, Wong SK, Berne MA (2003). Angiotensin-converting enzyme 2 is a functional receptor for the SARS coronavirus. Nature.

[39] Hersh EV, Wolff M, Moore PA, Theken KN, Daniell H (2022). A pair of “ACEs”. J Dent Res.

[40] Sinha S, Cheng K, Schäffer AA, Aldape K, Schiff E, Ruppin E (2020). In vitro and in vivo identification of clinically approved drugs that modify ACE2 expression. Mol Syst Biol.

[41] Poduri R, Joshi G, Jagadeesh G (2020). Drugs targeting various stages of the SARS-CoV-2 life cycle: exploring promising drugs for the treatment of Covid-19. Cell Signal.

[42] Han DP, Penn-Nicholson A, Cho MW (2006). Identification of critical determinants on ACE2 for SARS-CoV entry and development of a potent entry inhibitor. Virology.

[43] Huang X, Pearce R, Zhang Y (2020). De novo design of protein peptides to block association of the SARS-CoV-2 spike protein with human ACE2. Aging.

[44] Wrapp D, Wang N, Corbett KS, Goldsmith JA, Hsieh CL, Abiona O (2020). Cryo-EM structure of the 2019-nCoV spike in the prefusion conformation. Science.

[45] Hsieh CL, Goldsmith JA, Schaub JM, DiVenere AM, Kuo HC, Javanmardi K (2020). Structure-based design of prefusion-stabilized SARS-CoV-2 spikes. Science.

[46] Shang J, Ye G, Shi K, Wan Y, Luo C, Aihara H (2020). Structural basis of receptor recognition by SARS-CoV-2. Nature.

[47] Lan J, Ge J, Yu J, Shan S, Zhou H, Fan S (2020). Structure of the SARS-CoV-2 spike receptor-binding domain bound to the ACE2 receptor. Nature.

[48] Cantuti-Castelvetri L, Ojha R, Pedro LD, Djannatian M, Franz J, Kuivanen S (2020). Neuropilin-1 facilitates SARS-CoV-2 cell entry and infectivity. Science.

[49] Daly JL, Simonetti B, Klein K, Chen KE, Williamson MK, Antón-Plágaro C (2020). Neuropilin-1 is a host factor for SARS-CoV-2 infection. Science.

[50] Zalpoor H, Akbari A, Samei A, Forghaniesfidvajani R, Kamali M, Afzalnia A (2022). The roles of Eph receptors, neuropilin-1, P2X7, and CD147 in COVID-19-associated neurodegenerative diseases: inflammasome and JaK inhibitors as potential promising therapies. Cell Mol Biol Lett.

[51] Wang S, Qiu Z, Hou Y, Deng X, Xu W, Zheng T (2021). AXL is a candidate receptor for SARS-CoV-2 that promotes infection of pulmonary and bronchial epithelial cells. Cell Res.

[52] Bohan D, Ert HV, Ruggio N, Rogers KJ, Badreddine M, Aguilar Briseño JA (2021). Phosphatidylserine receptors enhance SARS-CoV-2 infection: AXL as a therapeutic target for COVID-19. bioRxiv.

[53] Morell AG, Gregoriadis G, Scheinberg IH, Hickman J, Ashwell G (1971). The role of sialic acid in determining the survival of glycoproteins in the circulation. J Biol Chem.

[54] Hoober JK (2020). ASGR1 and its enigmatic relative, CLEC10A. Int J Mol Sci.

[55] Gu Y, Cao J, Zhang X, Gao H, Wang Y, Wang J (2022). Receptome profiling identifies KREMEN1 and ASGR1 as alternative functional receptors of SARS-CoV-2. Cell Res.

[56] Zhang Q, Xiang R, Huo S, Zhou Y, Jiang S, Wang Q (2021). Molecular mechanism of interaction between SARS-CoV-2 and host cells and interventional therapy. Signal Transduct Target Ther.

[57] Li D, Edwards RJ, Manne K, Martinez DR, Schäfer A, Alam SM (2021). In vitro and in vivo functions of SARS-CoV-2 infection-enhancing and neutralizing antibodies. Cell.

[58] Bloch EM, Shoham S, Casadevall A, Sachais BS, Shaz B, Winters JL (2020). Deployment of convalescent plasma for the prevention and treatment of COVID-19. J Clin Invest.

[59] Shen C, Wang Z, Zhao F, Yang Y, Li J, Yuan J (2020). Treatment of 5 critically ill patients with COVID-19 with convalescent plasma. JAMA.

[60] Simonovich VA, Burgos Pratx LD, Scibona P, Beruto MV, Vallone MG, Vázquez C (2021). A randomized trial of convalescent plasma in Covid-19 severe pneumonia. N Engl J Med.

[61] Li L, Zhang W, Hu Y, Tong X, Zheng S, Yang J (2020). Effect of convalescent plasma therapy on time to clinical improvement in patients with severe and life-threatening COVID-19: a randomized clinical trial. JAMA.

[62] Bégin P, Callum J, Jamula E, Cook R, Heddle NM, Tinmouth A (2021). Convalescent plasma for hospitalized patients with COVID-19: an open-label, randomized controlled trial. Nat Med.

[63] RECOVERY Collaborative Group (2021). Convalescent plasma in patients admitted to hospital with COVID-19 (RECOVERY): a randomised controlled, open-label, platform trial. Lancet..

[64] Hueso T, Pouderoux C, Péré H, Beaumont AL, Raillon LA, Ader F (2020). Convalescent plasma therapy for B-cell-depleted patients with protracted COVID-19. Blood.

[65] Li W, Chen C, Drelich A, Martinez DR, Gralinski LE, Sun Z (2020). Rapid identification of a human antibody with high prophylactic and therapeutic efficacy in three animal models of SARS-CoV-2 infection. Proc Natl Acad Sci USA.

[66] Li W, Schäfer A, Kulkarni SS, Liu X, Martinez DR, Chen C (2020). High potency of a bivalent human V(H) domain in SARS-CoV-2 animal models. Cell.

[67] Hansen J, Baum A, Pascal KE, Russo V, Giordano S, Wloga E (2020). Studies in humanized mice and convalescent humans yield a SARS-CoV-2 antibody cocktail. Science.

[68] Baum A, Fulton BO, Wloga E, Copin R, Pascal KE, Russo V (2020). Antibody cocktail to SARS-CoV-2 spike protein prevents rapid mutational escape seen with individual antibodies. Science.

[69] Weinreich DM, Sivapalasingam S, Norton T, Ali S, Gao H, Bhore R (2021). REGN-COV2, a neutralizing antibody cocktail, in outpatients with Covid-19. N Engl J Med.

[70] Deeks ED (2021). Casirivimab/imdevimab: first approval. Drugs.

[71] CORIMUNO-19 Collaborative group (2022). Sarilumab in adults hospitalised with moderate-to-severe COVID-19 pneumonia (CORIMUNO-SARI-1): an open-label randomised controlled trial. Lancet Rheumatol.

[72] RECOVERY Collaborative Group (2021). Tocilizumab in patients admitted to hospital with COVID-19 (RECOVERY): a randomised, controlled, open-label, platform trial. Lancet..

[73] Rosas IO, Bräu N, Waters M, Go RC, Hunter BD, Bhagani S (2021). Tocilizumab in hospitalized patients with severe Covid-19 pneumonia. N Engl J Med.

[74] Chamlagain R, Shah S, Sharma Paudel B, Dhital R, Kandel B (2021). Efficacy and safety of sarilumab in COVID-19: a systematic review. Interdiscip Perspect Infect Dis.

[75] Park YJ, De Marco A, Starr TN, Liu Z, Pinto D, Walls AC (2022). Antibody-mediated broad sarbecovirus neutralization through ACE2 molecular mimicry. Science.

[76] Jing W, Procko E (2021). ACE2-based decoy receptors for SARS coronavirus 2. Proteins.

[77] Khan A, Benthin C, Zeno B, Albertson TE, Boyd J, Christie JD (2017). A pilot clinical trial of recombinant human angiotensin-converting enzyme 2 in acute respiratory distress syndrome. Crit Care.

[78] Monteil V, Kwon H, Prado P, Hagelkrüys A, Wimmer RA, Stahl M (2020). Inhibition of SARS-CoV-2 infections in engineered human tissues using clinical-grade soluble human ACE2. Cell.

[79] Feng F, Chen J, Zhao J, Li Y, Li M, Sun C (2021). Killing two birds with one stone by administration of soluble ACE2: a promising strategy to treat both cardiovascular diseases and SARS-CoV-2 infection. Viruses.

[80] Xiao T, Lu J, Zhang J, Johnson RI, McKay LGA, Storm N (2021). A trimeric human angiotensin-converting enzyme 2 as an anti-SARS-CoV-2 agent. Nat Struct Mol Biol.

[81] Tanaka S, Nelson G, Olson CA, Buzko O, Higashide W, Shin A (2021). An ACE2 triple decoy that neutralizes SARS-CoV-2 shows enhanced affinity for virus variants. Sci Rep.

[82] Chan KK, Dorosky D, Sharma P, Abbasi SA, Dye JM, Kranz DM (2020). Engineering human ACE2 to optimize binding to the spike protein of SARS coronavirus 2. Science.

[83] Sims JJ, Greig JA, Michalson KT, Lian S, Martino RA, Meggersee R (2021). Intranasal gene therapy to prevent infection by SARS-CoV-2 variants. PLoS Pathog.

[84] Chan KK, Tan TJC, Narayanan KK, Procko E (2021). An engineered decoy receptor for SARS-CoV-2 broadly binds protein S sequence variants. Sci Adv.

[85] Liu P, Wysocki J, Souma T, Ye M, Ramirez V, Zhou B (2018). Novel ACE2-Fc chimeric fusion provides long-lasting hypertension control and organ protection in mouse models of systemic renin angiotensin system activation. Kidney Int.

[86] Daniell H, Nair SK, Esmaeili N, Wakade G, Shahid N, Ganesan PK (2021). Debulking SARS-CoV-2 in saliva using angiotensin converting enzyme 2 in chewing gum to decrease oral virus transmission and infection. Mol Ther.

[87] Cao L, Goreshnik I, Coventry B, Case JB, Miller L, Kozodoy L (2020). De novo design of picomolar SARS-CoV-2 miniprotein inhibitors. Science.

[88] Linsky TW, Vergara R, Codina N, Nelson JW, Walker MJ, Su W (2020). De novo design of potent and resilient hACE2 decoys to neutralize SARS-CoV-2. Science.

[89] Shang J, Wan Y, Luo C, Ye G, Geng Q, Auerbach A (2020). Cell entry mechanisms of SARS-CoV-2. Proc Natl Acad Sci USA.

[90] de Vries RD, Schmitz KS, Bovier FT, Predella C, Khao J, Noack D (2021). Intranasal fusion inhibitory lipopeptide prevents direct-contact SARS-CoV-2 transmission in ferrets. Science.

[91] Outlaw VK, Bovier FT, Mears MC, Cajimat MN, Zhu Y, Lin MJ (2020). Inhibition of coronavirus entry in vitro and ex vivo by a lipid-conjugated peptide derived from the SARS-CoV-2 spike glycoprotein HRC domain. MBio.

[92] Hoffmann M, Kleine-Weber H, Schroeder S, Krüger N, Herrler T, Erichsen S (2020). SARS-CoV-2 cell entry depends on ACE2 and TMPRSS2 and is blocked by a clinically proven protease inhibitor. Cell.

[93] Breining P, Frølund AL, Højen JF, Gunst JD, Staerke NB, Saedder E (2021). Camostat mesylate against SARS-CoV-2 and COVID-19-rationale, dosing and safety. Basic Clin Pharmacol Toxicol.

[94] Hoffmann M, Hofmann-Winkler H, Smith JC, Krüger N, Arora P, Sørensen LK (2021). Camostat mesylate inhibits SARS-CoV-2 activation by TMPRSS2-related proteases and its metabolite GBPA exerts antiviral activity. EBioMedicine.

[95] Gunst JD, Staerke NB, Pahus MH, Kristensen LH, Bodilsen J, Lohse N (2021). Efficacy of the TMPRSS2 inhibitor camostat mesilate in patients hospitalized with Covid-19—a double-blind randomized controlled trial. EClinicalMedicine.

[96] Self WH, Semler MW, Leither LM, Casey JD, Angus DC, Brower RG (2020). Effect of hydroxychloroquine on clinical status at 14 days in hospitalized patients with COVID-19: a randomized clinical trial. JAMA.

[97] Ou T, Mou H, Zhang L, Ojha A, Choe H, Farzan M (2021). Hydroxychloroquine-mediated inhibition of SARS-CoV-2 entry is attenuated by TMPRSS2. PLoS Pathog.

[98] Ylä-Anttila P (2021). Autophagy receptors as viral targets. Cell Mol Biol Lett.

[99] Bosaeed M, Mahmoud E, Alharbi A, Altayib H, Albayat H, Alharbi F (2021). Favipiravir and hydroxychloroquine combination therapy in patients with moderate to severe COVID-19 (FACCT trial): an open-label, multicenter, randomized, controlled trial. Infect Dis Ther.

[100] Sivapalan P, Ulrik CS, Lapperre TS, Bojesen RD, Eklöf J, Browatzki A (2022). Azithromycin and hydroxychloroquine in hospitalised patients with confirmed COVID-19: a randomised double-blinded placebo-controlled trial. Eur Respir J.

[101] Omrani AS, Pathan SA, Thomas SA, Harris TRE, Coyle PV, Thomas CE (2020). Randomized double-blinded placebo-controlled trial of hydroxychloroquine with or without azithromycin for virologic cure of non-severe Covid-19. EClinicalMedicine.

[102] Sedighiyan M, Abdollahi H, Karimi E, Badeli M, Erfanian R, Raeesi S (2021). Omega-3 polyunsaturated fatty acids supplementation improve clinical symptoms in patients with Covid-19: a randomised clinical trial. Int J Clin Pract.

[103] Singh BO, Moirangthem B, Panda PK, Bahurupi YA, Saha S, group SCt (2021). Safety and efficacy of antiviral therapy alone or in combination in COVID-19—a randomized controlled trial (SEV COVID trial). medRxiv.

[104] Abd-Elsalam S, Soliman S, Esmail ES, Khalaf M, Mostafa EF, Medhat MA (2021). Do zinc supplements enhance the clinical efficacy of hydroxychloroquine? A randomized, multicenter trial. Biol Trace Elem Res.

[105] Angelini MM, Akhlaghpour M, Neuman BW, Buchmeier MJ (2013). Severe acute respiratory syndrome coronavirus nonstructural proteins 3, 4, and 6 induce double-membrane vesicles. MBio.

[106] Snijder EJ, Limpens R, de Wilde AH, de Jong AWM, Zevenhoven-Dobbe JC, Maier HJ (2020). A unifying structural and functional model of the coronavirus replication organelle: tracking down RNA synthesis. PLoS Biol.

[107] Ogando NS, Dalebout TJ, Zevenhoven-Dobbe JC, Limpens R, van der Meer Y, Caly L (2020). SARS-coronavirus-2 replication in Vero E6 cells: replication kinetics, rapid adaptation and cytopathology. J Gen Virol.

[108] Saraste J, Prydz K (2021). Assembly and cellular exit of coronaviruses: hijacking an unconventional secretory pathway from the pre-Golgi intermediate compartment via the Golgi ribbon to the extracellular space. Cells.

[109] Sannerud R, Marie M, Nizak C, Dale HA, Pernet-Gallay K, Perez F (2006). Rab1 defines a novel pathway connecting the pre-Golgi intermediate compartment with the cell periphery. Mol Biol Cell.

[110] Agostini ML, Andres EL, Sims AC, Graham RL, Sheahan TP, Lu X (2018). Coronavirus susceptibility to the antiviral remdesivir (GS-5734) is mediated by the viral polymerase and the proofreading exoribonuclease. MBio.

[111] Eastman RT, Roth JS, Brimacombe KR, Simeonov A, Shen M, Patnaik S (2020). Remdesivir: a review of its discovery and development leading to emergency use authorization for treatment of COVID-19. ACS Cent Sci.

[112] Kaka AS, MacDonald R, Greer N, Vela K, Duan-Porter W, Obley A (2021). Major update: remdesivir for adults with COVID-19: a living systematic review and meta-analysis for the American College of Physicians practice points. Ann Intern Med.

[113] Pan H, Peto R, Henao-Restrepo AM, Preziosi MP, Sathiyamoorthy V, Abdool Karim Q (2021). Repurposed antiviral drugs for Covid-19—interim WHO solidarity trial results. N Engl J Med.

[114] Beigel JH, Tomashek KM, Dodd LE, Mehta AK, Zingman BS, Kalil AC (2020). Remdesivir for the treatment of Covid-19—final report. N Engl J Med.

[115] Kabinger F, Stiller C, Schmitzová J, Dienemann C, Kokic G, Hillen HS (2021). Mechanism of molnupiravir-induced SARS-CoV-2 mutagenesis. Nat Struct Mol Biol.

[116] Cox RM, Wolf JD, Plemper RK (2021). Therapeutically administered ribonucleoside analogue MK-4482/EIDD-2801 blocks SARS-CoV-2 transmission in ferrets. Nat Microbiol.

[117] Fischer WA, Eron JJ, Holman W, Cohen MS, Fang L, Szewczyk LJ (2022). A phase 2a clinical trial of molnupiravir in patients with COVID-19 shows accelerated SARS-CoV-2 RNA clearance and elimination of infectious virus. Sci Transl Med..

[118] Báez-Santos YM, St John SE, Mesecar AD (2015). The SARS-coronavirus papain-like protease: structure, function and inhibition by designed antiviral compounds. Antivir Res.

[119] Clementz MA, Chen Z, Banach BS, Wang Y, Sun L, Ratia K (2010). Deubiquitinating and interferon antagonism activities of coronavirus papain-like proteases. J Virol.

[120] Owen DR, Allerton CMN, Anderson AS, Aschenbrenner L, Avery M, Berritt S (2021). An oral SARS-CoV-2 M(pro) inhibitor clinical candidate for the treatment of COVID-19. Science.

[121] Hammond J, Leister-Tebbe H, Gardner A, Abreu P, Bao W, Wisemandle W (2022). Oral nirmatrelvir for high-risk, nonhospitalized adults with Covid-19. N Engl J Med.

[122] Yuan Q, Liao Y, Torres J, Tam JP, Liu DX (2006). Biochemical evidence for the presence of mixed membrane topologies of the severe acute respiratory syndrome coronavirus envelope protein expressed in mammalian cells. FEBS Lett.

[123] Ghosh S, Dellibovi-Ragheb TA, Kerviel A, Pak E, Qiu Q, Fisher M (2020). β-Coronaviruses use lysosomes for egress instead of the biosynthetic secretory pathway. Cell.

[124] Liu X, Verma A, Garcia G, Ramage H, Lucas A, Myers RL (2021). Targeting the coronavirus nucleocapsid protein through GSK-3 inhibition. Proc Natl Acad Sci USA.

[125] Breitinger U, Ali NKM, Sticht H, Breitinger HG (2021). Inhibition of SARS CoV envelope protein by flavonoids and classical viroporin inhibitors. Front Microbiol.

[126] Park BK, Kim J, Park S, Kim D, Kim M, Baek K (2021). MERS-CoV and SARS-CoV-2 replication can be inhibited by targeting the interaction between the viral spike protein and the nucleocapsid protein. Theranostics.

[127] Siemieniuk RA, Bartoszko JJ, Díaz Martinez JP, Kum E, Qasim A, Zeraatkar D (2021). Antibody and cellular therapies for treatment of COVID-19: a living systematic review and network meta-analysis. BMJ.

[128] Siemieniuk RA, Bartoszko JJ, Ge L, Zeraatkar D, Izcovich A, Kum E (2020). Drug treatments for COVID-19: living systematic review and network meta-analysis. BMJ.

[129] Santos-Beneit F, Raskevicius V, Skeberdis VA, Bordel S (2021). A metabolic modeling approach reveals promising therapeutic targets and antiviral drugs to combat COVID-19. Sci Rep.

[130] You S, Li W, Guan Y (2018). Tunicamycin inhibits colon carcinoma growth and aggressiveness via modulation of the ERK-JNK-mediated AKT/mTOR signaling pathway. Mol Med Rep.

[131] Dawood AA, Alnori HA-M (2020). Tunicamycin anticancer drug may reliable to treat coronavirus disease-19. Open Access Maced J Med Sci.

[132] Bautista-Becerril B, Pérez-Dimas G, Sommerhalder-Nava PC, Hanono A, Martínez-Cisneros JA, Zarate-Maldonado B (2021). miRNAs, from evolutionary junk to possible prognostic markers and therapeutic targets in COVID-19. Viruses.

[133] Letafati A, Najafi S, Mottahedi M, Karimzadeh M, Shahini A, Garousi S (2022). MicroRNA let-7 and viral infections: focus on mechanisms of action. Cell Mol Biol Lett.

[134] Bartoszewski R, Dabrowski M, Jakiela B, Matalon S, Harrod KS, Sanak M (2020). SARS-CoV-2 may regulate cellular responses through depletion of specific host miRNAs. Am J Physiol Lung Cell Mol Physiol.

